# Glucocorticoids do not cause a clinically relevant elevation in the urine protein-to-creatinine ratio in dogs with inflammatory protein-losing enteropathy

**DOI:** 10.3389/fvets.2025.1751769

**Published:** 2026-01-23

**Authors:** Cameron J. Wood, Peter S. Chapman, Cara Horowitz

**Affiliations:** 1 BluePearl Veterinary Partners, Levittown, PA, United States; 2 Pacific Veterinary Emergency and Specialty Hospital, Lafayette, CA, United States

**Keywords:** canine, enteropathy, glucocorticoid, proteinuria, urine

## Abstract

**Objective:**

This study aimed to document changes in the urine protein-to-creatinine ratio (UPC) in dogs with inflammatory protein-losing enteropathy (iPLE) undergoing glucocorticoid therapy and with no overt evidence of concurrent renal disease.

**Methods:**

Dogs with histologically confirmed iPLE, a serum albumin level of <2.0 g/dL, gastrointestinal signs for ≥2 weeks, and no recent glucocorticoid use were prospectively enrolled at any of the four referral centers between 24 December 2020 and 25 May 2023. Dogs with azotemia, hepatopathy, urinary sediment abnormalities, or confirmed intestinal parasites were excluded. All dogs received prednisone or prednisolone (1 mg/kg BID), clopidogrel, and a new hypoallergenic or low-fat diet. Repeat urinalysis, UPC measurement, urine culture, blood pressure measurement, and serum chemistry analysis were performed after 1–2 weeks (T1) and 2–3 months (T2). Canine chronic enteropathy clinical activity index (CCECAI) scores were assessed at T0 and T1 for correlation with UPC.

**Results:**

Twelve dogs with iPLE were enrolled. Eight dogs were excluded due to comorbidities, loss to follow-up, or euthanasia. The median UPC increased from baseline (0.10) to T1 (0.25) and T2 (0.30). No dog had a UPC of >0.9. Mean CCECAI scores decreased from baseline (8.75) to T1 (3.08). UPC correlated with CCECAI at baseline [*ρ* (95%CI) 0.71 (0.22–0.91)], but not at follow-up [T1: *ρ* (95%CI) 0.04 (−0.55–0.60)] or changed from T0 to T1 [*ρ* (95%CI) 0.15 (−0.67–0.46)].

**Conclusion:**

Glucocorticoids increase the UPC in dogs with iPLE to the same extent as has previously been shown in healthy dogs.

**Clinical relevance:**

Clinically significant proteinuria in iPLE dogs treated with glucocorticoids should prompt clinicians to consider alternative etiologies.

## Introduction

Systemic glucocorticoid administration can induce proteinuria (increased urine-to-creatinine ratio, UPC), along with glomerular morphological changes, consistent with a steroid-associated glomerulopathy, in healthy dogs (1–3). The mechanism is suspected to be linked to hemodynamic changes, including an increased glomerular filtration rate (GFR) and blood pressure. These differences could influence both GFR and response to glucocorticoid administration in non-healthy dogs. Dogs with Systemic inflammatory response syndrome (SIRS) and sepsis have noted increases in UPC from systemic disease, along with dysregulation of cortisol-to-cortisone conversion (4, 5). Alternatively, studies have shown that a change in UPC may decrease following glucocorticoid administration due to improved management of the underlying condition, such as in dogs with immune-mediated polyarthritis (6).

Protein-losing enteropathy (PLE) is a complex syndrome that develops secondary to enteric mucosal compromise and loss of plasma proteins into the gastrointestinal lumen in some dogs with gastrointestinal disease (7, 8). Common diseases associated with canine PLE include chronic inflammatory enteropathies (CIE), intestinal lymphangiectasia (IL), intestinal neoplasia, or lymphangitis (7). Regardless of the underlying etiology, the prognosis is guarded, with disease-associated death occurring in up to 54% of cases (8). Although iPLE is considered to be a more chronic condition, patients often present with acute decompensation with severe hypoalbuminemia, cavitary effusions, peripheral edema, and a high risk of thromboembolic disease or death (9, 10). Severe hypoalbuminemia (concentration <2.0 g/dL) is a marker for mortality risk in these cases (7, 10).

Immunosuppressive glucocorticoids are sometimes used as a component of the treatment for unstable dogs with CIE-related PLE, but, as previously noted, glucocorticoids induce clinically significant renal proteinuria in healthy dogs (2, 3, 11). Proteinuria of various degrees has also been identified in several systemic inflammatory conditions, such as inflammatory bowel disease (IBD), immune-mediated hemolytic anemia (IMHA), immune-mediated polyarthritis (IMPA), acute pancreatitis, pyometra, and discospondylitis (6, 11). This effect might confound the interpretation of the significance of proteinuria in glucocorticoid-treated dogs with inflammatory PLE (iPLE) due to systemic illness, hospitalization, and concurrent drug effects on UPC.

The objective of this study was to describe changes in the urine protein-to-creatinine ratio (UPC) during glucocorticoid therapy in dogs with iPLE and with no overt evidence of concurrent renal disease.

## Methods

This study was approved by the BluePearl Science Veterinary Clinical Studies Committee (Study ID BPS0132) on 26 October 2020.

Dogs presented to any one of the four veterinary referral hospitals for suspected iPLE, as indicated by a baseline serum albumin of < 2.0 g/dL (RI 2.7–3.9 g/DL), globulins of < 2.5 (RI 2.0–3.6 g/dL), and a history of weight loss, diarrhea, hyporexia/anorexia, or vomiting for more than 2 weeks, between 24 December 2020 and 25 May 2023, were screened for prospective enrollment. The screening included a full abdominal ultrasound examination, a complete blood count, a serum biochemistry profile, a urinalysis with an UPC and an aerobic culture, a systolic blood pressure measurement, and fecal testing (flotation or antigen test) or an empiric treatment with fenbendazole (50 mg/kg daily for 5 days). Urine samples were uniformly collected via ultrasound-guided cystocentesis. Resting serum cortisol levels or Adrenocorticotropic Hormone (ACTH) stimulation testing, along with pre- and post-prandial bile acid testing, were performed at the discretion of the attending clinician. All dogs were evaluated with a gastroduodenoscopy with the collection of endoscopic biopsies of the gastric and duodenal mucosa using cup biopsy forceps. Dogs were not required to have a concurrent colonoscopy performed at the same time as gastroduodenoscopy to be included in this study.

Dogs were eligible if they met the above criteria and had a histopathologic diagnosis of lymphoplasmacytic enteritis (with or without lymphangiectasia) confirmed. Dogs were excluded from enrollment if they had an initial UPC of >0.2, abnormal renal or hepatic ultrasound findings, azotemia or abnormal liver enzymes on initial serum chemistry analysis, active urine sediment or a positive urine culture with evidence of pyuria or lower urinary tract clinical signs, or a positive fecal zinc sulfate centrifugation or antigen immunoassays. All urine samples underwent microscopic sediment examination, and samples demonstrating an active urine sediment—defined as >5 red blood cells/HPF, >5 white blood cells/HPF, or the presence of bacteria with lower urinary tract signs—were excluded from UPC analysis. Any changes to the urinary bladder on ultrasound imaging, suggesting inflammatory or infectious cystitis, also resulted in exclusion. Indirect arterial blood pressure was measured using a veterinary-specific oscillometric device (SunTech Vet 25, SunTech Medical, Morrisville, NC, United States) (18). Appropriately sized cuffs provided by the manufacturer were selected such that the cuff width was approximately 30–40% of the circumference of the limb. Dogs were positioned in lateral recumbency, and blood pressure measurements were obtained from the upward-facing pelvic limb. Dogs were allowed to acclimate to this position for at least 5 min prior to collecting results, and a minimum of five consecutive measurements were obtained for each dog. The mean of three to five consistent values was used for analysis. Dogs with systolic blood pressure between 160 and 180 mmHg were evaluated for evidence of stress-induced hypertension (which is also called “white coat syndrome”) based on concurrent assessments of heart rate, respiratory rate, and behavioral signs of anxiety in each subject. Animals with blood pressure results in this range and concurrent UPC values that were above the upper quartile (Q3) range were excluded from the analysis to avoid confounding the effects of hypertension with proteinuria. Dogs were also excluded if they had already received glucocorticoids or other immunosuppressive medications within 2 weeks of diagnosis.

On the day of enrollment (T0), dogs were prescribed prednisone or prednisolone (1 mg/kg PO q 12 h), clopidogrel (1–3 mg/kg PO q 24 h), and a strict hypoallergenic or low-fat diet following biopsy collection. Dogs already on a prescription diet prior to enrollment had a new diet provided, given a presumed failed response (n = 6). Diet choice was left to clinician preference and included commercial therapeutic hydrolyzed protein, commercial low-fat, commercial hybrid low-fat and hydrolyzed, commercial selected protein, and home-cooked ultra-low-fat. Although some studies have shown that an increasing number of dogs with iPLE can respond favorably to diet alone (12), such an approach is generally inadvisable if the serum albumin concentration is less than 2.0 g/dL, as dogs tend to be sicker, have a poor appetite, and rapidly declining albumin levels may worsen during the time required to implement a diet trial (10). Urinalysis, UPC, urine culture, blood pressure, and serum biochemistry were each evaluated after 1–2 weeks (T1) and at 2–3 months (T2). Additional oral immunosuppressive medications (e.g., chlorambucil or modified cyclosporine) could be added to the dog’s therapeutic protocol following T1 sample collection at the discretion of the managing clinician if the dog was clinically deteriorating.

Clinical severity of the disease was measured using the canine chronic enteropathy clinical activity index (CCECAI) at T0 and T1, which was also compared to UPC levels at these time points. The CCECAI is a standardized scoring system based on a combination of clinical parameters used to assess disease severity (19). The same questionnaire was filled out by owners at T0 and T1 to acquire the CCECAI scoring data. The CCECAI scores were not determined at T2 due to variability in treatment protocols once secondary immunosuppressive agents were allowed.

### Statistical analysis

An *a priori* power analysis was conducted using G*Power 3.1.9.6. To detect a mean difference of 0.5 in UPC values between T0 and T1 with 80% power, a two-sided paired t-test with a significance level of 0.05 was used. The assumptions included standard deviations of 0.1 (T0) and 0.6 (T1), a reference group size of 10 dogs, and a low within-dog correlation (r = 0.25), resulting in a required sample size of 12 dogs.

All statistical analyses were performed using the Statistical Analysis System (SAS) 9.4 (SAS Institute, Cary, NC, USA). Statistical significance was defined as a *p*-value of < 0.05. Data distribution was assessed through visual inspection of Q–Q plots, P–P plots, histograms, and skewness values. Normally distributed data were summarized using means and standard deviations (SDs), while non-normally distributed data were reported as medians with interquartile ranges (IQRs).

To evaluate the effect of glucocorticoid administration on UPC, linear mixed-effects models were used to compare post-treatment values to baseline. The model included a fixed effect for time and a random intercept for each dog. The assumptions of residual normality and homoscedasticity were assessed via conditional Q–Q plots, histograms, and residual plots. Log transformation of UPC values was applied to satisfy model assumptions. Dunnett’s adjustment was used for multiple comparisons. Satterthwaite’s approximation was used for degrees of freedom, and restricted maximum likelihood (REML) was used for parameter estimation.

Given the small sample size, Spearman’s correlation coefficients between UPC and CCACEI values were calculated using Fisher’s Z-transformation with bias correction to estimate confidence intervals.

## Results

Twenty dogs were screened over a period of 30 months. Two dogs were removed from the study during the initial screening due to a hookworm (Ancylostoma sp.) infection (*n* = 1) and one due to a histopathologic diagnosis of small cell alimentary lymphoma (*n* = 1). Six dogs failed to complete the study after enrollment due to an *E. coli*-positive urinary tract infection (UTI) (*n* = 1), repeatable hypertension (*n* = 1), loss to follow-up (*n* = 2), or euthanasia due to a poor response to treatment (*n* = 2). Twelve dogs meeting the inclusion criteria with confirmed iPLE secondary to IBD were enrolled and completed the study. Of the 12 dogs enrolled, 9 were direct transfers from emergency services due to acute decompensation because of clinical signs. All dogs were diagnosed via endoscopic biopsies and histopathology changes consistent with moderate to severe lymphoplasmacytic enteritis, with 9 out of 12 (75%) having only gastroduodenoscopy performed and 3 out of 12 (25%) having both gastroduodenoscopy and ileocolonoscopy performed. Specific biopsy findings included lamina propria lymphocytes and plasma cells (*n* = 12), lacteal dilation (*n* = 9), crypt distention (*n* = 8), lamina propria eosinophils (*n* = 8), intraepithelial lymphocytes (*n* = 7), lamina propria neutrophils (*n* = 6), villus stunting (*n* = 6), and epithelial injury (*n* = 5). These findings are comparable to previous histopathologic findings in dogs with PLE (Table 1) (13). Six dogs had crypt proteinosis and eosinophilic debris, cryptitis, and crypt rupture (previously referred to as crypt abscesses). Two dogs were allowed to remain in the study despite a diagnosis of subclinical bacteriuria (*E. coli*, one at T1 and one at T2). These dogs had no clinical signs of lower urinary tract infection, pyuria, or unexpected changes in UPC (Table 2).

**Table 1:** Histopathologic scores for intestinal biopsy specimens (modified WSAVA criteria).

Case no.	Crypt distension	Intraepithelial lymphocytes	Lacteal dilatation	LP eosinophils	LP lymphocytes or plasma cells	LP neutrophils	Mucosal fibrosis	Villous epithelial injury	Villous stunting
1	2	0	2	1	3	1	0	1	0
2	2	0	0	2	2	0	0	2	2
3	1	0	1	1	2	0	0	0	1
4	2–3	0	0	1	2	2	0	1	1
5	2	2	2	1–2	1–2	0	0	0	0
6	0	2	1	0	1	2	0	1	0
7	0	1	2	0	1	0	0	0	0
8	1	0	2	0	2	1	0	1	2
9	0	1	2	1	2	0	0	0	0
10	1	2	1	0	2	1	0	0	0
11	2	1	2	1	2–3	1	0	0	2
12	0	1–2	0	1	2	0	0	0	1

Histologic scoring: 0 = normal, 1 = mild, 2 = moderate, 3 = marked. LP = lamina propria.

**Table 2:** Individual clinicopathologic results for dogs with iPLE treated with glucocorticoids over time.

Case number	ALB (g/dL) T0	T1	T2	CRE (mg/dL) T0	T1	T2	Urea (mg/dL) T0	T1	T2	USG T0	T1	T2	BP (mmHg) T0	T1	T2	UPC T0	T1	T2
1	1.8	2.2	3.0	0.6	0.4	0.4	9	19	15	1.016	1.050	1.036	140	172	150	0.1	0.7	0.2
2	1.8	2.6	2.7	0.8	0.4	0.3	24	13	14	1.034	1.012	1.030	178	168	168	0.1	0.2	0.5*
3	1.7	2.5	3.0	1.2	0.8	0.8	28	29	29	1.054	1.049	1.049	115	138	153	0.1	0.3	0.9
4	1.8	3.1	3.7	0.6	0.5	0.4	12	26	22	1.009	1.021	1.012	116	114	127	0.1	0.1	0.2
5	1.5	2.4	2.7	0.9	0.5	0.6	12	13	15	1.034	1.011	1.026	114	133	124	0.1	0.2	0.2
6	1.3	1.8	2.9	0.7	0.4	0.4	10	9	12	1.021	1.018	1.024	136	121	112	0.2	0.5*	0.3
7	1.3	2.7	3.6	1.2	0.8	1.1	10	12	15	1.013	1.014	1.018	100	105	152	0.1	0.1	0.1
8	1.2	2.0	2.3	0.6	0.5	0.4	11	9	12	1.031	1.029	1.031	120	95	140	0.2	0.2	0.3
9	1.9	3.4	3.4	0.6	0.3	0.5	3	11	13	1.019	1.024	1.019	130	180	160	0.2	0.3	0.5
10	1.3	2.4	2.1	0.5	0.5	0.4	6	11	12	1.015	1.034	1.011	160	146	180	0.2	0.5	0.3
11	1.6	2.9	3.1	0.9	0.5	0.8	21	27	20	1.055	1.009	1.022	145	139	152	0.1	0.3	0.1
12	1.3	2.4	2.0	0.8	0.4	0.3	13	13	7	1.038	1.038	1.006	145	174	160	0.1	0.2	0.5

T0 = initial enrollment, T1 = 1–2 weeks post-treatment, T2 = 2–3 months post-treatment, ALB = albumin, CRE = creatinine, USG = urine-specific gravity, BP = systolic blood pressure, UPC = urine protein-to-creatinine ratio, * = positive urine culture for *E. coli*.

The mean (range) age of the dogs was 7.7 years (4.5–11.2). There were three Cocker Spaniel-Poodle crosses (25%), two Yorkshire Terriers (17%), two mixed-breed dogs (17%), and one (8%) each of English Bulldog, Rottweiler, Miniature Pinscher, Boston Terrier, and Maltese. There were six (50%) castrated males and six (50%) spayed females.

UPCs at T1 with a median (IQR) of 0.25 (0.20–0.40) and T2 with a median (IQR) of 0.30 (0.20–0.50) were significantly higher (T1 *p* = 0.004, T2 *p* = 0.001) than UPCs at T0 with a median (IQR) of 0.10 (0.10–0.20). There was a significant reduction in the mean CCECAI score following initiation of therapy, with mean CCECAI results decreasing from 8.75 to 3.08 (*p* < 0.001).

There was a significant correlation between UPC and CCECAI at baseline (rho [95% CI] = 0.71 [0.22–0.91], *n* = 12, *p* = 0.006); however, this association was no longer present at T1 (rho [95% CI] = 0.04 [−0.55–0.60], *n* = 12, *p* = 0.90), nor was there a significant correlation between changes in UPC and changes in CCECAI from T0 to T1 (rho [95% CI] = −0.15 [−0.67–0.46], *n* = 12, *p* = 0.64). CCECAI data were not collected at T2, given the variability in the treatment protocol.

The mean (SD) albumin and globulin levels at T0 were 1.5 g/dL (0.3; reference interval 2.7–3.9 g/dL) and 1.9 (0.5; reference interval 2.0–3.6), respectively. All dogs had a serum albumin level greater than 2.0 g/dL at T1 (Table 2). The mean time to reach this goal was 11 days (range 7–14), but it was dependent on scheduled recheck times. The mean (SD) albumin levels at T1 and T2 were 2.5 (0.4) and 2.9 (0.5), respectively. The mean systolic blood pressure measurement increased after starting glucocorticoids (T0 = 133 mmHg, T1 = 140 mmHg, T2 = 148 mmHg). Six dogs were given prednisone, and six were given prednisolone as therapy. The mean (SD) dose of glucocorticoids at T0 and T1 was 1.9 mg/kg/day (0.6). The timeframe to start tapering from this dose of glucocorticoids was variable, with a median time of 3.5 weeks. The mean (SD) dose of glucocorticoids at T2 was 1.0 mg/kg/day (0.7) with high variability from dog to dog (range 0–2.3 mg/kg/day), based on response to treatment. The mean (SD) clopidogrel dose administered was 2.4 mg/kg (0.68) once daily. This dose was not changed throughout the study unless discontinued. Eight dogs received additional immunosuppressive therapies, including four dogs treated with modified cyclosporine (mean [SD] 7.1 mg/kg/day [1.7]), three dogs treated with chlorambucil (mean [SD] 3.7 mg/m^2^/day [0.3]), and one dog treated with azathioprine (1.3 mg/kg/day).

Eleven of the 12 (92%) dogs that completed the study were alive after their 6-month follow-up. One dog (8%) was euthanized 3 months after starting treatment due to a poor response to therapy and presumed thromboembolic disease, despite receiving immunosuppressive prednisone, clopidogrel, a cobalamin supplement, modified cyclosporine, and a hydrolyzed low-fat diet.

## Discussion

Both exogenous glucocorticoid administration and endogenous hypercortisolism (e.g., hyperadrenocorticism) can induce proteinuria in dogs (1, 2, 14). Administration of oral prednisone (2.2 mg/kg q 12 h) and hydrocortisone (8 mg/kg q 12 h) in healthy dogs increases UPC (2, 3), but this study is the first to describe UPC dynamics in dogs treated with glucocorticoids for iPLE.

No dog in our study exhibited a UPC of >0.9. Additionally, 10 out of 12 dogs (83%) maintained a UPC of ≤ 0.5 at both T1 and T2, which is considered to be absent or borderline proteinuria based on International Renal Interest Society (IRIS) guidelines (15). Clinical intervention for proteinuria would therefore not be recommended in the majority of the dogs in this study. Thus, persistently elevated proteinuria (e.g., a repeatable UPC > 0.9) in dogs receiving glucocorticoid therapy for iPLE should prompt clinicians to consider alternative or concurrent causes of proteinuria. In these cases, further evaluation of proteinuria with SDS-PAGE or a renal biopsy, or adjunctive treatment with angiotensin-converting enzyme (ACE) inhibitors or angiotensin receptor blockers (ARBs), may be warranted to mitigate proteinuria and protect renal function. True proteinuria due to glomerular disease is expected to result in the loss of high-molecular-weight (HMW) proteins (> 60 kDa) in the urine, while steroid-induced proteinuria is expected to have a predominance of low-molecular-weight (LMW) proteins (< 60 kDa) and an absence of albumin or immunoglobulin bands on SDS-PAGE. Dogs with primary renal proteinuria are typically managed with a combination of dietary modifications, renin-angiotensin-aldosterone system (RAAS) inhibition, blood pressure control, and adjunct antithrombotic therapy.

Two dogs remained enrolled in the study despite positive urine culture results; these dogs were classified as having subclinical bacteriuria, as they had no signs of lower urinary tract disease or pyuria on urinalysis. Dogs on oral glucocorticoids are expected to have similar results to dogs with excess endogenous glucocorticoids, and the prevalence of subclinical bacteriuria in our cohort (16.7%) aligned with previously reported rates in dogs with hyperadrenocorticism (16). These dogs’ UPC values were not statistical outliers. In contrast, the dog that presented with clinical signs of a urinary tract infection and pyuria on urinalysis had a markedly elevated UPC of 1.7 and was excluded. Although glucocorticoids are a recognized risk factor for both subclinical bacteriuria and urinary tract infection, the clinical significance of a positive urine culture should be assessed in conjunction with clinical signs and quantitative UPC data.

The CCECAI is a widely used and validated tool for assessing disease severity and monitoring clinical responses in dogs with chronic enteropathies, including iPLE. In this study, we observed a significant reduction in mean CCECAI scores following the initiation of standardized glucocorticoid therapy and diet change, reflecting clinical improvement over the short-term follow-up period. CCECAI scores have previously been shown to be a helpful indicator to differentiate which dogs may be more likely to respond to low-fat or ultra-low-fat diet trials alone. This study found that dogs with a CCECAI score of greater than 8 are less likely to be food-responsive and need other concurrent therapies (17). The average CCECAI in this study was 8.75, with 7 of 12 patients having a score of 8 or higher at diagnosis. Baseline UPC initially correlated positively with CCECAI, suggesting that more clinically severe disease may be associated with higher levels of proteinuria, but this relationship was not maintained at follow-up. This finding may indicate that the drivers of proteinuria after starting glucocorticoids differ from the clinical severity of intestinal disease itself and are more directly impacted by glucocorticoid impacts on GFR. Together, these results suggest that UPC and clinical severity should be interpreted as related but distinct markers when managing dogs with iPLE.

The present study has several limitations. Although an *a priori* power calculation was performed, the study included only 12 dogs, which limits the ability to generalize the findings to a broader population of dogs with iPLE. A control group of healthy dogs treated with glucocorticoid therapy at the same dose and for the same amount of time was not used, which limits the ability to isolate the effects of glucocorticoid administration on UPC. The study was unmasked, and prospective cohort enrollment relied on clinical judgment and strict inclusion criteria, potentially introducing selection bias. Given the severity of the illness and the variability in response to therapy associated with dogs with iPLE, several aspects of the diagnostic workup and treatment could not be standardized. Although all dogs were maintained on high-dose glucocorticoids until their first recheck, the time at which tapering was initiated was left to the clinician. The choice of secondary immunosuppressive therapy and when to initiate it were variable after T1 testing was complete, which may have impacted UPC results at T2. Diet choice was not standardized, but dogs were fed a strict low-fat, hydrolyzed/limited ingredient, or combination diet. Dogs were also allowed to be treated with medications outside the scope of this study, including antibiotics, probiotics, anthelmintics, or anti-nausea medications (i.e., maropitant). The severity of inflammation or concurrent histopathologic changes in gastrointestinal biopsies could not be compared with UPC results. Blood pressure measurements were performed in clinical settings, and despite protocol efforts, the “white coat effect” likely influenced the results. Since only dogs that survived and completed all follow-up time points were included in the final analysis, survivorship bias may have led to an underestimation of UPC changes in dogs with more severe disease or a poorer treatment response.

In conclusion, these findings provide a reference point for expected changes in UPC following corticosteroid initiation in dogs with iPLE. Awareness of typical UPC responses may help clinicians better differentiate steroid-associated effects from underlying renal or urinary pathology.

## Data Availability

The raw data supporting the conclusions of this article will be made available by the authors, without undue reservation.
